# Targeting RNA modifications with pharmacological agents: New frontiers in cancer therapy

**DOI:** 10.1002/cam4.6989

**Published:** 2024-03-28

**Authors:** Angel Guan, Justin J.‐L. Wong

**Affiliations:** ^1^ Epigenetics and RNA Biology Laboratory Charles Perkins Centre, University of Sydney Camperdown Australia; ^2^ Faculty of Medicine and Health University of Sydney Camperdown Australia

**Keywords:** cancer, cancer therapy, chemoresistance, N6‐methyladenosine (m6A), RNA modification

## Abstract

The N6‐methyladenosine (m6A) RNA modification has gained significant prominence as a new layer of regulatory mechanism that governs gene expression. Over the past decade, various m6A regulators responsible for introducing, eliminating, and recognising RNA methylation have been identified. Notably, these m6A regulators often exhibit altered expression patterns in cancer, occasionally offering prognostic value. Nonetheless, the complex roles of these regulators in human cancer pathology remain enigmatic, with conflicting outcomes reported in different studies.In recent years, a multitude of inhibitors and activators targeting m6A regulators have been reported. Several of these compounds have demonstrated promising efficacy in both in vitro and in vivo cancer models. These findings collectively underscore the dynamic landscape of m6A regulation in cancer biology, revealing its potential as a therapeutic target and prognostic indicator.

## Graphical abstract

The m6A methylation is catalysed by the writer complex and the demethylation is catalysed by the erasers. Different readers proteins determine the fate of m6A modification. These proteins have increasingly been recognised as targets for cancer therapy.

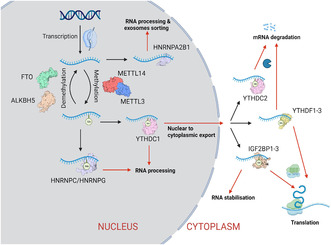

## 1 INTRODUCTION

Epitranscriptomics, the study of dynamic and reversible chemical modifications at the RNA level, plays a crucial role in understanding gene regulation.^1^ In mammals, N6‐methyladenosine (m6A) is the most prevalent internal RNA modification, with a frequency of 3–5 m6A per mRNA.^2^ Its abundance underscores its significance in normal physiology. Recent research indicates that aberrant m6A modification is linked to disease pathology, making it a potential therapeutic target.^3^

**FIGURE 1:**
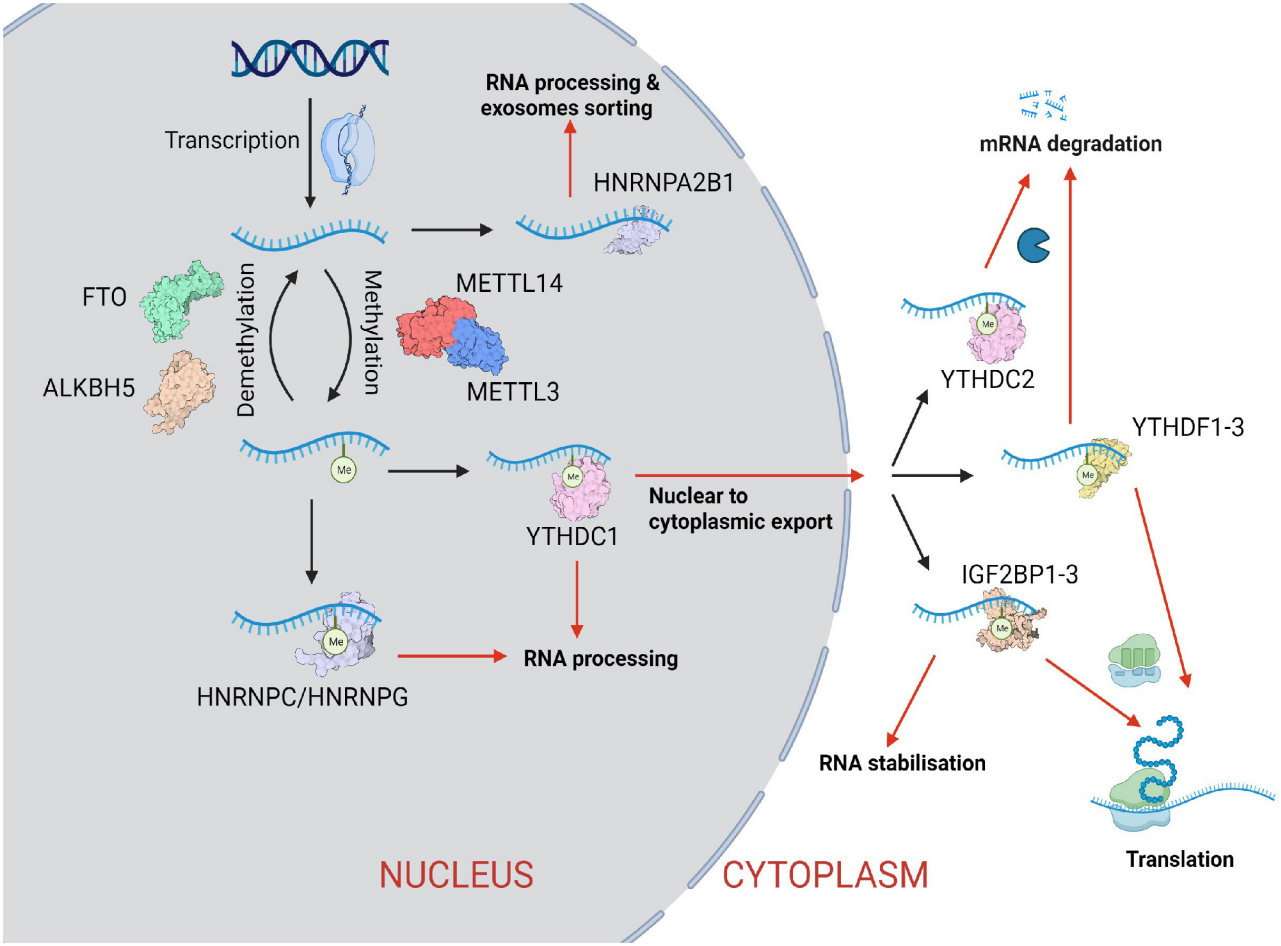
Mechanism of m6A modification regulating RNA metabolism. The m6A methylation is catalysed by the writer complex and the demethylation is catalysed by the erasers. Different readers proteins determine the fate of the RNA modification.

Three categories of proteins dynamically and reversibly regulate the RNA m6A modification process, affecting RNA biogenesis, stability, nuclear‐cytoplasmic export, translation, and splicing^4^ (Figure 1). m6A writers are methyltransferases that add a methyl group onto the target adenosine in RNA. m6A erasers are demethylases that remove the methyl group from the N6‐methyladenosine. m6A readers are RNA‐binding proteins that recognise the m6A‐marked RNA, determining the functional outcome of the m6A modification.^4^ This review summarises the known m6A regulators and briefly discusses their often‐opposing roles in promoting or perturbing cancer progression, chemotherapeutic resistance, and immunotherapy. We also provide an up‐to‐date review of potential therapies targeting m6A modification.

### 1.1 The m6A writers, erasers and readers

Before the last decade, studying m6A modification was limited due to the lack of sequencing techniques, quantitative strategies and knowledge concerning proteins that regulate m6A.^5^ The catalytic component of the m6A writer, Methyltransferase‐Like Protein 3 (METTL3), was identified in 1997, 25 years after the first discovery of m6A modification in 1974.^6^, ^7^ Methyltransferase‐Like Protein 14 (METTL14) was then reported as the second protein in the m6A writer complex, functioning together with METTL3 to enhance its activity.^8^ Completing the core m6A‐methyltransferase complex, Wilms's tumour 1‐associating protein (WTAP) interacts with the METTL3/METTL14 to allow their localisation into nuclear speckles.^9^ Other cofactors include VIRMA (KIAA1429), ZC3H13, HAKAI (CBLL1), RBM15 and RBM15B.^10^, ^11^, ^12^ VIRMA mediates mRNA m6A modification in 3'UTR and near stop codon, explaining the enrichment of m6A modification at these specific regions of mRNAs.^11^, ^13^ ZC3H13 interacts with WTAP, bridging the METTL3/METTL14/WTAP complex to the other cofactors, which is also essential for localising the complex.^12^, ^14^ HAKAI is essential for the stabilisation of core components of the complex where disruption of HAKAI leads to degradation of VIRMA and ZC3H13.^15^, ^16^ RBM15 and RBM15B have redundant functions in interacting with WTAP, responsible for guiding the complex to the specific RNA target, XIST.^10^ They bind U‐rich regions of the RNA,^10^ but whether they are essential for all m6A modification by the METTL3/METTL14 complex remains elusive.

Other m6A writers described act on RNA species other than mRNAs. METTL16 installs m6A on U6 snRNA and S‐adenosylmethionine (SAM) synthetase pre‐mRNA.^17^ While various ncRNAs, lncRNA, and pre‐mRNA were reported to associate with METTL16, whether all these interactions involved m6A methylation demand further research.^18^ Recently, METTL5‐TRMT112 complex and ZCCHC4 were reported to methylate 18S and 28S rRNA, respectively.^19^, ^20^, ^21^

Two established m6A erasers are Fat mass and obesity‐associated protein (FTO) and alkB homologue 5 RNA demethylase (ALKBH5). FTO is associated with human obesity and energy homeostasis, demethylating m6A in cellular mRNA and other RNA species.^22^, ^23^ ALKBH5, on the other hand, specifically demethylates m6A‐marked mRNA or sometimes m6A‐marked ssDNA, showing minimal activity towards m6A rRNA or other types of RNA modifications.^24^ The substrate specificity of FTO and ALKBH5 is influenced by the conformational diversity of RNA, determined by both the sequence and the conformational changes due to m6A modification.^25^

The functional outcome of an m6A modification is predominantly determined by the m6A‐binding proteins that ‘read’ the modification, including the YTH domain‐containing proteins (YTHDC1‐2), the YTH domain family (YTHDF1‐3), the insulin‐like growth factor 2 mRNA binding proteins (IGF2BP1–3), and the HNRNP family (HNRNPA2B1, HNRNPC, HNRNPG).

Nuclear m6A readers regulate alternative splicing and nuclear‐cytoplasmic export of RNA. YTHDC1 selectively targets GG(m6A)CU over GA(m6A)CU^26^ and recruits the splicing factor SRSF3 to promote exon inclusion. YTHDC1 also promotes nuclear‐cytoplasmic export by recruiting NXF1.^27^ Other nuclear readers involved in pre‐RNA processing are HNRNPC and HNRNPG.^28^, ^29^ However, the mechanism of regulation and how they select their targets remain elusive. HNRNPA2B1 binds m6A‐marked primary microRNAs and promotes miRNA processing and exosome sorting.^30^ Interestingly, HNRNPA2B1 has a greater affinity to non‐methylated RNA than methylated RNA, suggesting a potential ‘m6A‐switch’ mechanism for regulating RNA metabolism rather than an ‘m6A‐promoting’ mechanism.^31^

Cytoplasmic m6A readers regulate RNA stability and translation. Only simultaneous knockout of YTHDF1‐3 impair RNA degradation, suggesting they redundantly mediate RNA degradation.^32^, ^33^, ^34^ YTHDC2 also promotes RNA degradation.^35^ In contrast, IGF2BPs promote RNA stability and translation.^36^ YTHDF1 and YTHDF3 enhance mRNA translation by interacting with translation initiation factors, including eIF3, eIF4A3, and eIF4A3.^37^, ^38^ However, neither simultaneous nor independent knockout of YTHDF1‐3 reduces the translation efficiency, suggesting that they are not regulating translation in cells at homeostasis.^34^ While the role of YTHDFs in mRNA translation remains controversial, it appears that they may be associated with stress granule formation and possibly regulate m6A‐associated translation of a limited number of mRNAs under stressful conditions in physiological and pathological conditions, including cancer.^39^, ^40^

## 2 THE COMPLEX ROLE OF m6A REGULATORS AS ONCOPROTEINS AND TUMOUR SUPPRESSORS

Given their key roles in normal physiology, m6A writers, erasers, and readers have been implicated in diverse human cancers. Notably, the m6A regulators often display opposing roles as oncoproteins and tumour suppressors (Tables 1, 2, 3). In this section, we will discuss the complex role of some m6A regulators in tumorigenesis, leading us to explore the prospects of potential therapeutic approaches in the following section.

**TABLE 1:** Complex roles of aberrant m6A writers' expression in human cancers.

m6A regulators	Tumour suppressor gene(TSG)/Oncogene	Downstream target RNA	Cancer type	References
METTL14	Oncogene	MYB, MYC	AML	[69,204,205]
	Oncogene	CXCR4, CYP1B1		
	Oncogene	AC084125.2	Breast cancer	[54,53,206]
	Oncogene	miR‐146a‐5p		
	TSG	APC	TNBC, ER‐, PR‐breast cancer	[207]
	TSG	PHLPP2, mTORC2	Endometrial cancer	[208]
	TSG	XIST	CRC	[69, 70, 71, 72]
	TSG	ARRDC3		
	TSG	Wild Type P53		
	TSG	SOX4		
METTL3	Oncogene	c‐MYC, BCL2, PTEN	AML	[41, 42, 43]
	Oncogene	SP1		
	Oncogene	MDM2		
	TSG	COL3A1	TNBC	[45, 46]
	Oncogene	KRT7	Breast cancer	[48, 49, 50, 51, 52, 209, 210, 211]
	Oncogene	EZH2		
	Oncogene	Bcl‐2/Oncogene		
	Oncogene	SOX2, CD133, CD44		
	Oncogene	LATS1		
	Oncogene	lncRNA MALAT1		
	Oncogene	CCNE1	CRC	[59, 60, 61, 62, 63, 64, 65, 66, 67, 68]
	Oncogene	HK2, SLC2A1		
	Oncogene	MYC		
	Oncogene	SOX2		
	Oncogene	YPEL5		
	Oncogene	SOCS2		
	Oncogene	CRB3		
	Oncogene	pri‐miRNA‐196b		
	Oncogene	miR‐1246		
	Oncogene	SOX2, SOX4, EZH2, ADAM19,MGMT, APNG, SRSF, MYC	Glioblastoma	[55, 56, 58, 212, 213]
METTL5	Oncogene		Lung adenocarcinoma	[214]
	Oncogene	c‐MYC	Hepatocellular carcinoma	[76]
	TSG		Gastric cancer	[77]
	Oncogene		Breast cancer	[73]
	Oncogene	18S rRNA	HCC	[215]
	Oncogene		UCEC	[75]
	Oncogene	18S rRNA	Nasopharyngeal carcinoma (NPC)	[216]
	Oncogene	c‐MYC	Pancreatic cancer	[74]
METTL16	Oncogene	GPX4	Breast cancer	[78]
	Oncogene	Cyclin D1	Gastric cancer	[79]
	Oncogene	eIF4E2	Lung cancer	[80]
	TSG	TME	Pancreatic ductal adenocarcinoma	[217]
	Oncogene	BCAT1, BCAT2	AML	[81]
	TSG		Endocrine system tumours	[83]
	Oncogene	lncRNA RAB11B‐AS1	HCC	[82]

**TABLE 2:** Complex roles of aberrant m6A erasers' expression in human cancers.

m6A regulator	Tumour suppressor gene(TSG)/Oncogene	Downstream target RNA	Cancer type	References
FTO	Oncogene	PDGFB/ERK pathway	NPM1‐mutated AML	[90]
	Oncogene	ASB2, RARA	MLL‐rearranged AML	[89]
	Oncogene	TP53INP2	NPM1‐mutated AML	[88]
	Oncogene	miR‐181b‐3p	HER+ Breast cancer	[86]
	Oncogene	BNIP3	Breast cancer	[84, 85]
	Oncogene	5't‐RF‐GlyGCC > FTO, reduce eIF4G1, inhibit autophagy, promote progression		
	Oncogene	MZF1, c‐MYC	CRC	[218, 219, 220, 221]
	Oncogene	MYC		
	Oncogene	G6PD, PARP1		
	TSG	MTA1		
	Oncogene	AKT	Ovarian Cancer	[93, 222]
	TSG			
	TSG	SNAI1	Epithelial ovarian cancer	[92]
	TSG	PDE1C, PDE4B (blocking cAMP signalling)	Ovarian Cancer	[91]
ALKBH5	TSG	SLC7A11	CRC	[100, 101, 102, 103]
	TSG	FOXO3		
	TSG	PHF20		
	Oncogene	SAV1	MM	[223]
	Oncogene	lncRNA NEAT1, EZH2	Gastric cancer	[224, 225, 226, 227]
	TSG	SLC7A2, CGB3		
	TSG	PKMYT1		
	Oncogene	JAK1		
	TSG	YAP	NSCLC	[98]
	TSG	TIAM1	Thyroid cancer	[99]
	Oncogene	FOXM1	Glioblastoma	[94]
	Oncogene	TACC3	AML	[96]
	Oncogene	ITPA	t(8:21) AML	[95]
	Oncogene	AXL	AML	[97]

**TABLE 3:** Complex roles of aberrant m6A readers expression in human cancers.

m6A reader	Tumour suppressor gene(TSG)/Oncogene	Target RNA	Cancer type	References
YTHDF1	Oncogene	Cyclin E2	AML	[228]
	Oncogene	ANLN	Hepatocellular carcinoma	[109, 110, 229]
	Oncogene	ATG2A&ATG14		
	Oncogene	PI3K/AKT/mTOR pathway		
	Oncogene	E2F8	Breast cancer	[48, 104, 105, 129]
	Oncogene	PKM2		
	Oncogene	KRT7		
	Oncogene	FOXM1		
	Oncogene	EGFR	Intrahepatic cholangiocarcinoma	[230]
	Oncogene	PLK1, PI3K/AKT pathway	Prostate cancer	[231]
	Oncogene	ARHGEF2	CRC	[232, 233]
	Oncogene	SH3TC2		
	Oncogene	CNOT7	Osteosarcoma	[234]
	TSG	HINT2	Oscular melanoma	[235]
	Dependent	Snail	Gastric cancer	[236, 237]
	Oncogene	USP14		
YTHDF2	Oncogene	EGR1/p21cip1/waf1/CDK2‐cyclin E1 pathway	Multiple myeloma	[238, 239]
	Oncogene	STAT5A/MAP2K2/p‐ERK pathway		
	Oncogene	TME‐RIG‐I(TSG)	Bladder cancer	[240]
	Dependent	HIF1α/CBSLR/YTHDF2/CBS pathway	Gastric cancer	[241, 242, 243]
	TSG	FOXC2		
	TSG	PPP2CA		
	Oncogene	lncRNA FENDRR	Endometrial cancer	[244, 245]
	Oncogene	CDKN1B	Intrahepatic cholangiocarcinoma	[130]
	Oncogene	AXIN	Cervical cancer	[131]
	Oncogene	circ_SFMBT2	NSLCL	[246]
	Oncogene	mTOR/AKT	Lung squamous cell carcinomas	[247]
	Oncogene	FGF14‐AS2(decay)/RUNX2	Breast cancer	[108]
	Oncogene	AXIN	Lung Adenocarcinoma	[248, 249]
	Controversial	FAM83D‐TGFβ1‐SMAD2/3		
	Oncogene	UBXN1	Glioma	[250]
	Oncogene	LXRA&HIVEP2	Glioblastoma	[251]
	TSG	EGFR	Hepatocellular carcinoma	[112, 252, 253]
	Oncogene	Tnfrsf2	AML	[254]
	Oncogene		t(8;21)AML	[255]
YTHDF3	Oncogene	PFKL	HCC	[111]
	Oncogene	CTNNB1	Melanoma	[256, 257]
	Oncogene	LOXL3		
	Oncogene	PGK1	Osteosarcoma	[258]
	Oncogene	ZEB1	TNBC	[259]
	Oncogene		CRC	[260]
	Oncogene	ITGA6	Hepatocellular carcinoma	[261]
	Oncogene	EGFR, GJA1, ST6GALNAC5	Breast cancer	[106, 107, 262]
IGF2BP1	Oncogene	CTP1A	Breast cancer	[263]
	Oncogene	SRF, FOXM1, IQGAP3	Gastric cancer	[264]
	Oncogene	TK1	NSCLC	[265]
	Oncogene	EZH2	Neuroendocrine neoplasms (NENs)	[195]
	Dependent	c‐MYC	Breast cancer (Hypoxic refractory BC)	[266]
	Oncogene	PEG10	Endometrial cancer	[267]
	Dependent	c‐MYC	Highly metastatic cancer (Colorectal, breast, ovarian, nasopharyngeal)	[268]
	Oncogene	SRF, PDLIM7, FOXK1	HCC, EOC	[269]
	Oncogene	E2F	Pancreatic ductal adenocarcinoma	[197]
IGF2BP2	Oncogene	NOTCH1	T‐ALL	[138]

### 2.1 m6A writers: METTL3 and METTL14

METTL3 is overexpressed in Acute Myeloid Leukaemia (AML) cells compared to healthy haematopoietic cells. Promoter‐bound METTL3 promotes the translation of oncoproteins, including SP1, facilitating AML development.^41^ METTL3's tumorigenic effect involves widespread mRNA targets, including c‐MYC, BCL2, PTEN, and MDM2.^42^, ^43^ Similarly, METTL14 is significantly upregulated in AML carrying t(11q23), t(15;17), or t(8;21).^44^ Mechanistically, METTL14 increases m6A levels on the MYB and MYC transcripts, preventing cell differentiation but enhancing survival and proliferation.^44^

Conversely, METTL3 and METTL14 act as tumour suppressors in Triple‐Negative Breast Cancer (TNBC), where their downregulation leads to tumour growth and metastasis. Mechanistically, METTL3 is repressed by miR‐34c‐3p in TNBC,^45^ and it negatively regulates the expression of the oncogenic COL3A1.^46^ METTL3 depletion also contributes to tumour progression in Hormone Receptor Positive (HR+) and Human Epidermal Growth Factor Receptor 2 Negative (HER2‐) breast cancer.^47^ However, the roles of METTL14 and METTL3 in breast cancer, in general, are controversial. Despite the tumour‐suppressive functions evidenced in the experimental studies on TNBC and HR + HER2‐ breast cancer, other studies have reported the oncogenic roles. METTL3 enhances the stability and translation efficiency of oncoproteins, Bcl‐2, SOX2, CD133, CD144, EZH2, and KRT7, or reduces the stability of tumour suppressor gene, LATS1.^11^, ^48^, ^49^, ^50^, ^51^, ^52^ These studies covered a wide range of breast cancer subtypes including TNBC, HER2+, and HR+ breast cancer. METTL14 was also reported to promote breast cancer cell proliferation, migration and invasion by methylating miRNAs.^53^, ^54^ The complex role of the methyltransferase complex in breast cancer indicates a need for further exploration of how m6A affects carcinogenesis in subtype‐specific breast cancer.

Overexpression of METTL3 in glioblastoma stem cells (GSCs) has been correlated with a poor prognosis for glioblastoma and its silencing in GSCs has been shown to reduce tumour growth in vivo.^55^ Multiple studies consistently highlight that inhibiting METTL3 not only diminishes the self‐renewal capacity of GSCs but also increases their sensitivity to Temozolomide (TMZ) treatment and radiotherapy, both in vitro and in vivo.^55^, ^56^, ^57^, ^58^ Mechanistically, METTL3 stabilises DNA repair genes MGMT and APNG, thereby enhancing sensitivity to chemotherapy with TMZ.^58^ Additionally, it stabilises SOX2, facilitating SOX2‐dependent DNA repair during radiotherapy.^55^

In colorectal cancer (CRC), upregulated METTL3 is associated with a poor prognosis and promotes cellular proliferation and metastasis in vitro and in vivo. Examples of downstream RNA affected are CCNE1 which regulates the cell cycle,^59^ HK2 and SLC2A1 which are involved in glycolysis,^60^, ^61^ and SOX2 and MYC which interact with key proliferative pathways such as EGFR, Akt, NOTCH and Wnt signalling.^62^, ^63^ Notably, METTL3 also decreases the stability or translation efficiency of tumour‐suppressor genes. Examples include YPEL5, SOCS2 and CRB3.^64^, ^65^, ^66^ METTL3 also targets ncRNAs, including pri‐miRNA, to regulate their processing, leading to aberrant expression of their cognate target oncogenes and tumour‐suppressor genes.^67^, ^68^

In CRC, loss of METTL14 is linked to an unfavourable prognosis and has been shown to increase cellular proliferation and invasion by regulating SOX4, and lncRNA XIST.^69^, ^70^, ^71^ The contrasting effects of METTL3 and METTL14 on CRC progression despite their complex formation and catalytic enhancement may be attributed to their preference for different targets, leading to diverse downstream pathways.^8^, ^71^ Moreover, the tumour‐suppressive role of METTL14 in p53‐wild‐type CRC cells, while not significantly affecting p53‐mutant or p53‐null CRC cells, highlights the influence of tumour heterogeneity on m6A regulators' roles.^72^ The observed controversies can be attributed, at least partially, to this heterogeneity. More examples of the complex roles of METTL3/METTL14 in diverse cancers are shown in Table 1.

### 2.2 Other m6A writers

The recently identified m6A writers, METTL5 and METTL16, have also been implicated in cancer (Table 1). METTL5 is overexpressed in breast cancer,^73^ pancreatic cancer,^74^ uterine corpus endometrial carcinoma,^75^ and hepatocellular carcinoma (HCC),^76^ but significantly decreased in gastric cancer tissues compared to adjacent normal tissues and intestinal metaplasia tissues.^77^ METTL16 facilitates the progression of breast,^78^ gastric,^79^ lung,^80^ AML,^81^ and liver cancers.^82^ Conversely, METTL6 expression is positively correlated with the overall survival of endocrine system tumours.^83^

### 2.3 m6A erasers: FTO and ALKBH5

The role of FTO in breast cancer is complex and contradictory. One on hand, FTO promotes breast cancer cell proliferation, colony formation, cellular invasion, and metastasis in vitro and in vivo.^84^, ^85^, ^86^ FTO demethylates BNIP3 mRNA and induces its degradation to inhibit apoptosis while increasing cell proliferation.^85^ Demethylation of m6A at miR‐181p‐3p by FTO inhibits the miRNA function to allow expression of the oncogenic ARL5B, promoting cellular invasion and migration.^86^ In this context, FTO inhibition could be a potential therapeutic strategy for breast cancer. Conflicting with the above, FTO downregulation was also reported in breast cancer, promoting tumour progression and metastasis via enhancing expression of mesenchymal markers including SNAI2, VIM, FN1, NT5E, SNAI1, MMP2 and ZEB1 while decreasing epithelial markers FSTL3, KRT18 and TJP1.^87^ Moreover, FTO‐depleted cells showed increased Wnt signalling and are sensitive to Wnt inhibitor therapy.^87^

FTO is overexpressed in specific subtypes of AML, including t(11q23)/MLL‐rearranged AML, t(15;17)/Acute Promyelocytic Leukaemia (APL), and normal karyotype AMLs carrying NPM1 or FLT3‐ITD mutants.^88^, ^89^, ^90^ In these cases, FTO overexpression leads to the downregulation of ASB2 and RARA proteins, promoting the overexpression of oncogenic MLL and the activation of the PDFGRB/ERK pathway.^89^ The presence of other markers, such as NPM1 mutation type A, would induce FTO expression, resulting in TP53INP2 upregulation which promotes autophagy and leukaemia cell survival.^88^, ^90^ Such mechanisms suggest potential correlations between m6A regulators and specific AML subtypes, highlighting the potential for precision treatments targeting m6A modifications in AML.

FTO is downregulated in ovarian cancer stem cells and tumours.^91^ Downregulation of FTO increases the m6A level in the SNAI1 transcript, enhancing its stability via an IGF2BPs‐dependent manner and promoting epithelial‐to‐mesenchymal transition.^92^ FTO inhibits ovarian cancer stem cell self‐renewal by upregulating PDE1C and PDE4B, which subsequently block the cAMP signalling pathway.^91^ However, FTO was also reported to be upregulated in ovarian tumour tissues, increasing cellular viability and autophagy function but decreasing apoptosis.^93^ Therefore, the role of FTO in ovarian cancer remains controversial, possibly due to the different cancer models. The mechanism of action of FTO in ovarian cancer demands further research.

Similarly, oncogenic and tumour‐suppressive roles of ALKBH5 have been reported. ALKBH5 enhances the expression of FOXM1 via demethylation, promoting stem‐like cell proliferation and tumourigenesis in glioblastoma.^94^ In AML, overexpressed ALKBH5 post‐transcriptionally reduces the stability of *TACC3*, *AXL* and *ITPA* transcripts to promote cancer stem‐cell self‐renewal.^95^, ^96^, ^97^ In contrast, ALKBH5 functions as a tumour suppressor in thyroid cancer and Non‐Small Cell Lung Cancer (NSCLC) by reducing the expression of *TIAM1* and *YAP*, respectively.^98^, ^99^ In CRC, downregulation of ALKBH5 is associated with poor prognosis.^100^, ^101^ Downstream transcripts were identified to be PHF20, FOXO3, and SLC7A11, in which the stability of PHF20 and SLC7A11 are decreased by ALKBH5 while the FOXO3 mRNA's stability is enhanced.^100^, ^102^, ^103^ Recently reported examples of FTO and ALKBH5‐mediated cancer development and progression are detailed in Table 2.

### 2.4 m6A readers

The role of m6A readers in cancer is also complex (Table 3). For example, the overexpression of all YTHDF1‐3 has been implicated in breast cancer progression and metastasis. In breast cancer, HIF1α induced by hypoxia inhibits miR‐16‐5p, which under normal conditions targets and inhibits YTHDF1 via mRNA 3′UTR.^104^ However, hypoxia‐induced YTHDF1 overexpression enhances the translation of PKM2 and subsequently upregulates glycolysis.^104^ YTHDF1 also upregulates the translation of oncogenic FOXM1.^105^ YTHDF3, a prognostic biomarker for breast cancer, promotes brain metastasis by enhancing the expression of key metastatic genes including GJA1, ST6GALNAC5, and EGF.^106^, ^107^ YTHDF2 mediates the m6A‐dependent degradation of the lncRNA FGF14‐AS2, and patients with high YTHDF2 and low FGF14‐AS2 expression have worse distant metastasis‐free survival.^108^

In HCC, YTHDF1 is upregulated by HIF1α under hypoxic conditions, facilitating the translation of autophagy‐related genes *ATG2A* and *ATG14* in an m6A‐dependent manner.^109^ YTHDF1 also positively regulates *ANLN*, promoting HCC bone metastasis.^110^ YTHDF3 is overexpressed in HCC and correlates with poor prognosis.^111^ While YTHDF3 is generally accepted to promote RNA degradation or enhance translation, it was found to stabilise *PFKL* mRNA, leading to increased expression and promoting aerobic glycolysis and carcinogenesis.^111^ In contrast, YTHDF2 is downregulated under hypoxic conditions,^112^ and forced YTHDF2 expression promotes the degradation of oncogenic *EGFR* mRNA, suppressing HCC cell proliferation and growth in vitro and in vivo.^112^ However, contrary to that study, other research has demonstrated that YTHDF2 is a negative downstream target of a frequently downregulated miRNA in HCC, miR‐145.^113^ Recently reported examples of m6A reader‐mediated cancer development and progression are detailed in Table 3.

## 3 M6A REGULATORS ARE KEY PLAYERS IN CHEMORESISTANCE

Chemoresistance remains a life‐threatening obstacle in cancer biology and clinical practice. Multiple factors and mechanisms have been identified, carrying important clinical implications. m6A regulators were linked to chemoresistance (Table 4), providing a potential combination therapeutic strategy.

**TABLE 4:** Examples of aberrant m6A regulators' expression in chemoresistance.

Protein	Promote/Prevent chemoresistance	Chemotherapy	Target RNA	Cancer type	References
METTL3	Promote	Platinum‐etoposide	DCP2	SCLC	[121]
	Promote	Doxorubicin or 5‐flurouracil (5‐FU)	p53 (R273H) mutant	Colon cancer	[122]
	Promote	Adriamycin	MALAT1	Breast cancer	[116]
	Prevent	Doxorubicin, paclitaxel and cisplatin	CDKN1A, BAX	Breast cancer	[47]
	Prevent	Daunorubicin, cytarabine	AKT1	AML	[114]
	Promote	Idarubicin	ITGA4 (increase)	AML	[120]
	Promote	Adriamycin	mi‐R221‐3p	Breast cancer	[270]
	Promote	Adriamycin	Promoted EGF expression	Breast cancer	[134]
	Promote	Docetaxel	LINC00662 and miR‐186‐5p	TNBC	[118]
	Promote	Oxaliplatin	TRAF5	CRC	[119]
FTO	Promote	Oxaliplatin		AML	[175]
	Promote	Doxorubicin		Breast cancer	[123]
	Promote	Pan‐resistant	IRX3	TNBC	[187]
	Promote	Gefinitib	MYC, BCRP, MRP7	Breast cancer, NSCLC	[176]
	Promote	Nilotinib	MerTK, Bcl‐2	AML	[185]
	Promote	Doxorubicin	ZEB1	Breast cancer	[271]
	Promote	5‐FU	SIVA1	CRC	[272]
	Prevent	Platinum, etoposide	NNMT	Ovarian cancer	[124]
ALKBH5	Promote	Temozolomide	SOX2	Glioblastoma	[125]
	Promote	Trastuzumab and lapatinib (Her2‐targeted therapy)	GLUT4	Breast cancer	[126]
	Prevent	Gemcitabine	WIF‐1	Adenocarcinoma	[127]
YTHDF1	Promote	Cisplatin	GLS1	CRC	[128]
	Promote	Adriamycin (DNA‐damaging chemotherapy) and Cisplatin as well as Olaparib, a PARP inhibitor.	E2F8	Breast cancer	[129]
YTHDF2	Promote	Cisplatin	AXIN1	Cervical cancer	[131]
	Promote	Cisplatin	CDKN1B	Intrahepatic cholangiocarcinoma (ICC)	[130]
YTHDF3	Promote	Oxaliplatin		CRC	[132]
	Promote	Radiotherapy	RAD51D	Cervical cancer	[134]
YTHDC2	Promote	Radiotherapy	IGF1R‐AKT/S6 signalling pathway.	Nasopharyngeal carcinoma (NPC).	[135]
YTHDC1	Prevent	Sunitinib	ANXA1‐MAPK pathway	ccRCC	[133]
IGF2BP1	Promote	Doxorubicin	ABCB1	Colorectal adenocarcinoma	[137]
	Promote	Doxorubicin	ERRα	Osteosarcoma (OS)	[136]
IGF2BP2	Promote	Cytarabine, dexamethasone, vincristine, and venetoclax.	NOTCH1	T‐ALL	[138]

Both upregulation and downregulation of METTL3 impact cancer sensitivity to chemotherapy, further highlighting the intricate role of m6A regulators. In breast cancer, METTL3 upregulation correlates with Adriamycin resistance and key downstream targets were identified to be MALAT1, EGF, and miR‐221‐3p.^114^, ^115^, ^116^ Downregulation of METTL3 has been reported in HR + HER2‐ breast cancer, promoting resistance to doxorubicin, paclitaxel, and cisplatin.^47^ These seemingly contradictory findings may arise from different downstream readers recognising the m6A‐marked mRNA. Despite similarities in DNA‐damaging chemotherapies' primary mode of action, each therapy targets multiple pathways providing additional effects and interactions with cells and the tumour microenvironment (TME). For instance, doxorubicin induces immunogenic cell death, stimulating immune responses, and inhibiting regulatory T cells.^117^ Thus, its anti‐tumour effect extends beyond DNA damage to immunomodulation. The complexity and the heterogeneity of chemotherapeutic response in different cancer subtypes contribute to these conflicting results.

METTL3 mediates resistance to other chemotherapeutic drugs, including platinum‐etoposide in Small Cell Lung Cancer (SCLC), doxorubicin, 5‐fluorouracil (5‐FU), and oxaliplatin in CRC, Idarubicin in AML, and docetaxel in breast cancer.^114^, ^118^, ^119^, ^120^, ^121^, ^122^ Downregulation of METTL3 also associates with resistance to daunorubicin and cytarabine in AML.^114^

The m6A erasers FTO and ALKBH5 also mediate chemoresistance in cancer. The upstream regulator STAT3 promotes FTO expression in breast cancer, resulting in doxorubicin resistance that can be reversed by FTO knockdown.^123^ FTO overexpression targets apoptosis‐inducing factor SIVA1, conferring 5‐FU‐resistance in CRC cells.^124^ Consistently, inhibition of FTO pharmacologically or genetically reduced the 5‐FU tolerance of CRC xenograft models.^124^ These results suggest that FTO inhibitors hold the potential for overcoming chemoresistance, which is discussed further in the below section. However, FTO also exhibits a protective role, its downregulation was found in platinum (Pt)‐resistant ovarian cancer cells and forced expression increases sensitivity to Pt in vitro and in vivo.^124^

ALKBH5 mediates Temozolomide resistance in glioblastoma by demethylating the SOX2 transcript, increasing its expression.^125^ In breast cancer, ALKBH5 demethylates GLUT4 mRNA, enhancing its stability and correlating with resistance to trastuzumab and lapatinib.^126^ In addition, ALKBH5 targets the WIF‐1 transcript, enhancing its expression and activating Wnt signalling, resulting in gemcitabine resistance in adenocarcinoma.^127^

YTHDF1 overexpressed in cisplatin‐resistant CRC cells, promotes GLS1 protein expression, elevating glutamine metabolism and cisplatin resistance.^128^ YTHDF1 knockdown enhances sensitivity to Adriamycin, cisplatin, and Olaparib in breast cancer cells.^129^ YTHDF2 is also involved in cisplatin resistance, the key downstream targets were found to be AXIN1 in cervical cancer, and CDKN1B in intrahepatic cholangiocarcinoma.^130^, ^131^ YTHDF3 is highly expressed in oxaliplatin‐resistant CRC tissue, facilitating eIF2AK2 and eIF3A recruitment on mRNAs to regulate translation.^132^ In contrast, YTHDC1 is downregulated in clear cell renal cell carcinoma (ccRCC) and reduces sensitivity to sunitinib.^133^ Apart from chemoresistance, YTHDF3 and YTHDC2 correlate with radiotherapy resistance in cervical cancer and nasopharyngeal carcinoma. Mechanistically, YTHDF3 promotes RAD51D translation and YTHDC2 activates the IGF1R/ATK/S6 signalling axis, both in an m6A‐dependent manner.^134^, ^135^

The IGF2BPs also mediate chemoresistance. Overexpression of IGF2BP1 mediates doxorubicin resistance via stabilising the mRNA of oestrogen‐related receptor alpha (ERRα) and ABCB1.^136^, ^137^ Similarly, IGF2BP2 overexpression causes chemoresistance to cytarabine, dexamethasone, vincristine, and venetoclax in T‐cell acute lymphoblastic leukaemia (T‐ALL) by recognising m6A‐marked NOTCH1 mRNA and stabilising it.^138^

## 4 m6A REGULATORS ARE KEY PLAYERS IN CANCER IMMUNOLOGY

Cancer immunotherapy has revolutionised the cancer treatment in the last decade, with notable successes such as immune checkpoint blockades (ICBs) and CAR‐T cell therapy.^139^ The influence of m6A regulators extends beyond cancer cells to encompass immune cells within the TME, potentially influencing the outcomes of immunotherapies. Consequently, m6A regulators emerge as promising targets for combination therapy with ICBs or cell therapies, as detailed in Table 5.

**TABLE 5:** Examples of aberrant m6A regulators' expression in cancer immunology.

Protein	Promote/Prevent Immunotherapies resistance	Immunotherapies	Target RNA	Cancer type	References
METTL3	Prevent (m6a regulators + to help ICBs)	PD‐1 blockade	SPRED2	Orchestrates cancer	[143]
FTO	Promote	PD‐1 blockade	c‐Jun, C/EBPβ, JunB	Melanoma, CRC	[144]
	Promote	PD‐1 blockade	PD‐1, CXCR4, SOX10	Melanoma	[145]
ALKBH5	Promote	PD‐1 blockade	Mct4, Slc16a3	Melanoma, Colon cancer	[146]
	Controversial	PD‐1 blockade	PD‐L1	Intrahepatic cholangiocarcinoma	[147]
	Promote	PD1 blockade	AXIN2	CRC	[273]
YTHDF1/YTHDF2			PD‐L1	NSCLC	[150]
YTHDF2			PD‐1, TIM3, CTLA4	Lower‐grade glioma	[274]
YTHDF1	Promote	PD‐1 blockade	CXCL1	CRC	[148]
IGF2BPs	Promote	PD‐1 blockade		Melanoma	[275]

METTL3 suppresses anti‐tumour immune response by reducing granzyme B and interferon gamma‐positive CD8+ T cell infiltration.^140^ METTL3 depletion synergises with anti‐PD‐1 blockade, impeding tumour progression in various in vivo models, including CRC, melanoma, and HCC.^140^, ^141^ Recently, in vivo models demonstrated that METTL3 inhibition is equally efficacious to anti‐PD‐1 therapy and combination of both provide synergism.^142^ Mechanistically, catalytic inhibition of METTL3 results in dsRNA formation and potent cell‐intrinsic interferon responses that can stimulate anti‐tumour immunity, which is distinct to the mechanism of the current ICBs and cell therapy. Importantly, the combination of anti‐PD1 and METTL3 inhibitor can augment antitumor immunity to eliminate malignant clones insensitive to these agents alone, suggesting that METTL3 and ICBs work through distinct but complementary pathways.^142^

However, conflicting findings suggest that selective ablation of METTL3 in myeloid cells remodels the TME, increasing M1/M2‐like tumour‐associated macrophage and regulatory T (Treg) cell infiltration.^143^ Moreover, myeloid‐specific METTL3 depletion attenuates efficacy of anti‐PD‐1 in melanoma. This level of contradictory might attribute to the different functions of METTL3 in cancer and immune cells, underscoring the complexity of targeting METTL3 in cancer immunotherapy.

FTO‐mediated m6A demethylation, on the other hand, elevates the expression of transcription factors c‐Jun, JunB, and C/EBPβ, thereby enhancing glycolytic metabolism and inhibiting CD8+ T cell infiltration.^144^ Others immune related genes upregulated by FTO‐mediated demethylation includes PD‐1, CXCR4, and SOX10.^145^ Preclinical models of melanoma and CRC reveal synergism between FTO inhibition and anti‐PD‐1 therapy.^144^, ^145^ The m6A eraser ALKBH5, when deleted, sensitises tumours to ICBs in vivo.^146^ Mechanistically, ALKBH5 positively regulates Mct4/Slc16a3 and lactate levels during anti‐PD‐1/GVAX treatment, increasing Treg cells and myeloid‐derived suppressor cells (MDSCs) accumulation in TME. Moreover, lower ALKBH5 expression in melanoma correlates with better response to anti‐PD‐1 therapies such as pembrolizumab or nivolumab. However, the paradoxical role of ALKBH5 in different cancers is evident, as it positively regulates PD‐L1 expression in intrahepatic cholangiocarcinoma.^147^ Patients with strong nuclear expression patterns of ALKBH5 exhibit greater sensitivity to anti‐PD‐1 therapy, emphasising the diverse functions of m6A regulators across cancer types.

Similarly, the roles of m6A readers vary substantially in different cancer types. In CRC, YTHDF1 impairs anti‐tumour immunity by negatively regulating CD8+ T cell infiltration while upregulating CXCL1 to promote MDSCs infiltration.^148^ Consistently, YTHDF1 knockout increases anti‐PD1 efficacy and CD8+ infiltration in CRC.^148^, ^149^ However, almost all subsets of tumour‐infiltrating lymphocytes including CD8+ T cells are high in high YTHDF1 and YTHDF2 lung cancers, suggesting distinct downstream target genes of m6A readers between cancer types.^150^ Further examples of m6A readers influencing antitumor immunity are detailed in Table 5.

It is crucial to note that genetic knockout or siRNA‐mediated depletion may differ from pharmacological inhibition, which holds greater relevance in clinical applications. The subsequent section will explore the effects of inhibiting m6A regulators with small molecules in combination with immunotherapy.

## 5 THERAPEUTIC POTENTIAL

While the relationship between m6A and cancer has been extensively studied, the development of therapeutics targeting m6A regulators is still in its infancy.

### 5.1 M6A writer‐METTL3

The study of m6A modulators, including METTL3 inhibitors, has gained increasing attention due to their roles in regulating gene expression in cancer cells. Targeting METTL3 based on its diverse functions holds promise for developing precision cancer therapies (Table 6).

**TABLE 6:** Therapies targeting METTL3.

Mode of Action	Therapy	Anti‐tumour effect (In vitro)	Anti‐tumour effect (In vivo)	Disease	References
Competitive inhibitor	Cpb‐564			Renal injury and inflammation	[159]
	STM2457	Human AML cell lines treated with STM2457 shows significant growth reduction in a concentration‐dependent manner.	Treatment leads to impairment of engraftment and AML expansion in vivo and significantly prolongs the mouse lifespan.	AML	[153]
				Allograft rejection	[276]
		The IC50 values of cisplatin and etoposide are significantly decreased after treatment with STM2457.	Significantly inhibits the growth rate of the xenografts when combined with chemotherapy.	SCLC (METTL3 induce chemoresistance to the platinum‐etoposide therapy)	[121]
			Synergise with anti‐PD‐1	HCC	[140]
	STM3006	Induce interferon responses and enhance cytotoxic CD8+ T cells response.	No efficacy in vivo		[142]
	UZH1a	Reduces m6A/A ratio in MOLM‐13 and U2OS cell lines.		AML, osteosarcoma	[152]
	Analogues and derivatives of adenosines				[151]
	SANCDB0370, SANCDB0867, and SANCDB1033				[154]
	Quercetin	Efficiently inhibits the viability of MIA PaCa‐2 and Huh7 tumour cells.		Liver cancer, pancreatic cancer	[155]
Allosteric inhibitor	CDIBA	Shows dose‐dependent anti‐proliferative activities in multiple AML cell lines.		AML	[160]
	Eltrombopag	Shows anti‐proliferative in multiple AML cell lines, and demonstrates synergistic effect when combined with venetoclax and cytarabine.		AML	[161]
Activator	Piperidine derivative and piperazine derivative compounds	Shows proliferative effect and increases m6A level in HEK293 cells.			[162]

#### 5.1.1 Competitive inhibitors

METTL3 was extensively reported as an oncoprotein (Table 1); therefore, METTL3 inhibitors have the potential to be anti‐tumour drugs. The first reported METTL3 inhibitor, adenosine, competitively binds to the SAM binding site as METTL3 is an S‐adenosyl‐L‐methionine‐dependent methyltransferase.^151^ Subsequent docking studies of 4000 adenosine‐moiety‐containing compounds into the SAM binding site identified 70 hits.^151^ Experimental validation of these hits led to the discovery of 7 candidates with promising inhibitory effects. However, the anti‐tumour efficacy of these adenosine derivatives was not tested in cancer cell lines or mouse models. Furthermore, the selectivity of this class of inhibitors remains to be examined.

A structure‐based drug discovery approach led to the discovery of the potent and selective UZH1a.^152^ Co‐crystal of UZH1a‐METTL3 revealed a significant conformational rearrangement (6Å displacement) of the Lys513 side chain, distinguishing it from the Lys513 orientation observed in the co‐crystal structure of METTL3 with sinefungin, a non‐selective inhibitor of SAM‐dependent methyltransferases. This unique conformation of METTL3 induced by UZH1a is believed to contribute to its selectivity. Notably, UZH1a possesses favourable physicochemical properties, such as low molecular weight and good cellular permeability. Demonstrating high‐nanomolar potency in a biochemical assay, UZH1a effectively reduces the m6A/A ratio in the AML MOLM‐13 cell line and the osteosarcoma U1OS cell line. However, the anti‐tumour effect of UZH1a has yet to be tested in vitro or in vivo.

Through high‐throughput screening (HTS) of 250,000 compounds, STM2457 was identified as a potent (IC50 = 16.9 nM) and selective METTL3 inhibitor.^153^ STM2457 competitively binds the SAM binding pocket. Consistent with the oncogenic role of METTL3 in AML,^41^, ^42^, ^43^ treatment of mouse and human AML cells with STM2457 consistently demonstrates growth reduction, myeloid differentiation, and cell cycle arrest.^153^ Moreover, treatment with STM2457 induces apoptosis in human and mouse AML cell models but not in normal non‐leukaemic haematopoietic cells, which could be advantageous in minimising side effects. The result is replicable in in vivo model, expanding the lifespan of mice with minimal toxicity observed.^153^ In addition to the oncogenic role, METTL3 is responsible for SCLC chemoresistance and STM2457 successfully reversed the chemoresistant in vitro and in vivo.^121^

Very recently, the competitive inhibitor STM3006 was published.^142^ It has 20‐fold increased cellular potency compared with STM2457 and potently inhibits proliferation but induces apoptosis of multiple cell lines. While STM3006 is structurally distinct from STM2457, they have very similar binding poses revealed by x‐ray crystallography, possibly explaining the high selectivity of both inhibitors. In addition, STM3006 inhibition results in a cell‐intrinsic interferon response and enhanced antigen‐dependent tumour killing by cytotoxic CD8+ T cells. STM3006 has rapid metabolism and has no efficacy in vivo but its improved oral availability version, STC‐15, is now under phase I clinical trial in solid cancer (NCT05584111).

Virtual screening of 1012 South African natural products led to the identification of three candidates, SANCDB0370, SANCDB0867, and SANCDB1033, derived from *Buddleja salviifolia*, *Croton gratissimus*, and *Struthiola argentea*, respectively.^154^ These candidates exhibit more negative free energy than STM2457. The in silico analysis suggested that these compounds possess drug‐like properties and lower toxicity compared to STM2457.^154^ It is important to note that while the computational methods provided valuable insights into the candidates' properties, wet‐lab experiments are yet to be conducted to validate their activity in vitro or in vivo.

Quercetin, a competitive METTL3 inhibitor with micromolar potency, was recently discovered through the virtual screening of natural products.^155^ Quercetin is cell permeable and capable of decreasing mRNA m6A levels in human pancreatic adenocarcinoma cells.^155^ Quercetin's anti‐proliferative effects have been confirmed in various cancer cell lines, including liver, lung, breast, and pancreatic cancer cells.^155^ Notably, quercetin has been studied for its anti‐tumour properties for over two decades, and clinical trials have shown no reported toxicity or side effects.^156^, ^157^ However, quercetin is a non‐specific inhibitor with pleiotropic effects and can target multiple enzymes, including DNA methyltransferases and histone deacetylases.^158^ Despite its potential safety as a cancer patient supplement, the lack of selectivity for METTL3 suggests the need for further optimization. Virtual screening also revealed Cpd‐456, while it has demonstrated potential in protecting against renal injury and inflammation, its anti‐tumour efficacy remains unstudied.^159^

#### 5.1.2 Allosteric inhibitors

Besides competitive inhibitors, two allosteric inhibitors were found to inhibit METTL3. The first allosteric inhibitor, known as CDIBA, potently inhibits the METTL3/METTL14 complex but not the individual METTL3 and METTL14 subunits, indicating its simultaneous binding to METTL3 and METTL14.^160^ In contrast, Eltrombopag exhibits similar inhibitory activity on the complex and METTL3 alone but not on METTL14.^161^ Computational studies consistently suggested that the putative binding site of Eltrombopag is on the METTL3.^161^ Additionally, Eltrombopag selectively targets METTL3 over other histone methyltransferases, including DOT1L, G9a, PRMT1, SETD2, and SMYD3. Both CDIBA and Eltrombopag demonstrated an anti‐proliferative effect in the AML cell line MOLM‐13, leading to a reduction in the m6A level.^160^, ^161^

#### 5.1.3 METTL3 activator

Four potential METTL3 activators were identified via virtual screening.^162^ The series of compounds containing piperidine and piperazine rings showed high docking efficiencies. These compounds, which partially occupy the SAM pocket, seem to activate the methylation activity of METTL3/METTL14. The ability of these compounds to reactivate METTL3 to suppress cancer subtypes associated with METTL3 downregulation remains to be explored.

### 5.2 M6A eraser‐FTO

FTO has long been studied as a promising molecular target for treating obesity.^163^ Therefore, more inhibitors have been developed for FTO than for METTL3 (Table 7). These FTO inhibitors are primarily competitive, binding to either the substrate (i.e. methylated ssRNA/DNA) or the cofactor (i.e. 2‐oxoglutarate (2OG)) binding site. To the best of our knowledge, no allosteric inhibitor has been reported.

**TABLE 7:** Therapies targeting FTO and ALKBH5.

m6A regulator	Mode of action	Therapy	Anti‐tumour effect (In vitro)	Anti‐tumour effect (In vivo)	Disease	References
FTO	Competitive inhibitor	18077, 18097	18097 suppresses proliferation and increases the chemosensitivity of various cancer cells. It also inhibits in vitro invasion capability of cancer cells.	18097 significantly suppresses tumour growth in MDA‐MB‐231 xenograft models, and inhibits breast cancer lung metastasis.	Breast cancer	[170]
		CHTB			Obesity	[167]
		N‐CDPCB			Obesity	[165]
		Meclofenamic acid 2	Exerts a substantial inhibitory effect on the growth and the self‐renewal of various cancer cell lines.	PBT003‐grafted mice treated with MA2 has smaller tumour size with decreased tumour luciferase activity and prolonged survival.	Glioblastoma	[56, 174]
		MA	Shows significant synergistic effects with gefinitib in gefinitib‐resistant NSCLC cell lines.		Gefinitib‐resistant NSCLC	[175, 176]
		Rhein	Shows synergetic effect when combined with nilotinib or PKC412.	Nilotinib plus rhein significantly supresses tumour formation.	Leukaemia	[164, 175]
			Inhibits m6A demethylation.			
		13a	Inhibits colony formation of NB4 cells in concentration‐dependent manner.	Improves the survival rate of MONOMAC6‐transplanted NSG mice.	AML	[179]
		FB23 and FB23‐2	Suppresses proliferation and promotes the differentiation/apoptosis of human AML cells and primary blast AML cells.	Inhibits the progression of human AML cell lines and primary cells in xeno‐transplanted mice and shows prolonged survival.	AML	[178]
		R‐2HG	Exhibits a broad growth‐suppressive activity in leukaemia in general.	Treatment leads to decrease of leukaemic blasts in mice with sensitive cells.	Leukaemia and glioma	[183, 184]
			Glycolysis is suppressed by R‐2HG in sensitive leukaemia cells.		IDH‐wt and IDH‐mutant AML.	
		FTO‐4				[181]
		FTO‐43			AML/glioblastoma, Gastric cancer	[182]
		Quinolone derivatives				[169]
		GNPIPP12MA nanoparticles		The combination of GNPIPP12MA with PD‐L1 blockade significantly inhibits leukaemia progression and metastasis in a mouse model.		[180]
		Dac51	Enhances release of cytokines and cytotoxic capacity in T cells co‐cultured with Dac51‐pretreated B16OVA tumour cells.	Inhibited tumour growth in vivo.		[144]
		Entacapone			Prostate cancer	[168, 171, 172]
			Induces apoptosis (KYSE‐30: 50%. YM‐1:22.6%) and has a modulatory effect on cell cycle progression.		Oesophageal cancer	
		Saikosaponin D, Saikosaponin A	SsD supresses proliferation, colony formation ability but induces cell cycle arrest and apoptosis.	SsD inhibits lung/spleen metastasis and prolongs survival.	AML	[185]
		CS1 and CS2	CS1 suppresses CRC cell proliferation in 6 colorectal cancer cell lines and in the 5‐Fluorouracil resistant cell line.	CS1 suppresses in vivo tumour growth in the HCT116 heterotopic model.	CRC	[188, 189]
			Treatment leads to increased apoptosis and cell cycle arrest at the G0 phase in human AML cells.	CS2 reduces leukaemia infiltration and doubled the overall survival in the patient‐derived xenotransplantation AML model.	AML	
		MO‐I‐500	Inhibits survival and/or colony formation of SUM149‐MA cells.		Rare Panresistant Triple‐Negative Inflammatory Breast Cancer Cells	[186, 187]
	Activator	Tricyclic antidepressants: imipramine (IMI), imipramine (AMT)			Depression	[190]
ALKBH5	Competitive inhibitor	2‐[(1‐hydroxy‐2‐oxo‐2‐phenylethyl)sulfanyl]acetic acid, 4‐{[(furan‐2‐yl)methyl]amino}‐1,2‐diazinane‐3,6‐dione	Suppresses cell proliferation in some leukaemia cell lines.		AML	[277]
		ALK‐04		Reduces tumour growth in mice model.	Melanoma	[146]
		MV1035	Reduces U87 glioblastoma cell line migration and invasiveness.		Glioblastoma	[191]
		Ena21	Shows a dose‐dependent inhibition of cell growth in cell lines tested.		Glioblastoma Multiforme	[193]
	Uncompetitive inhibitor	Ena15				
		IOX1 (Non‐specific inhibitor targeting most 2‐OG oxygenase)		Synergise with anti‐PD‐1	Glioblastoma	[278, 279, 280]
		20 m				[192]

#### 5.2.1 Competitive inhibitors

Via virtual screening, rhein and N‐CDPCB were identified as competitive FTO inhibitors, increasing the m6A level in cells.^164^, ^165^ Molecular modelling revealed that rhein binds to m3T, 2OG, and Fe2+ binding sites, disrupting the cofactor and substrate binding.^166^ Similarly, N‐CDPCB binds to the substrate‐binding site by occupying the space between an antiparallel sheet and the extended C‐terminal of the long loop of FTO.^165^ Given that the loop of FTO is not conserved in other mammalian ALKB members, N‐CDPCB is likely to be a selective inhibitor.^165^ Another competitive inhibitor, CHTB, also binds to the non‐conserved site of FTO, suggesting good selectivity.^167^ In the absence of experimental validation, the selectivity profile of these competitive inhibitors remains largely unknown. Furthermore, the anti‐tumour efficacy of N‐CDPCB and CHTB has not been tested.

More FTO inhibitors were discovered through structure‐based virtual screening, encompassing entacapone from FDA‐approved drugs, two quinolone derivatives from the ZINC library, and 18,077 and 18,079 from the commercial database Specs (https://www.specs.net).^168^, ^169^, ^170^ Entacapone, an FDA‐approved therapy for Parkinson's disease in combination with levodopa and carbidopa was found to induce apoptosis in oesophageal cancer cell lines YM‐1 and KYSE‐30.^171^, ^172^ The two quinolone derivatives, identified as FTO inhibitors, were originally investigated for supporting the survival of dopamine neurons in neurodegenerative disease and their anti‐tumour efficacy requires further studies.^169^ In vitro studies demonstrated that 18,097 significantly suppresses the colony number of cancer cells.^170^ Moreover, 18,097 enhances the sensitivity of HeLa cervical cancer cells and MDA‐MB‐231 breast cancer cells to cisplatin and doxorubicin. It also inhibits cancer cell invasion by downregulating the expression of matrix metalloproteinase 2 (MMP2), fibronectin (FN), and vimentin.^170^

Meclofenamic acid (MA) is a nonsteroidal anti‐inflammatory drug approved by the FDA in 1980, commonly used to treat pain and inflammation associated with osteoarthritis, rheumatoid arthritis, and menstrual cramps.^173^ In 2015, it was found to selectively inhibit FTO's demethylation activity compared to other ALKB Family members.^174^ A prodrug called MA2 has also been developed, featuring an extra ethyl ester group to increase cell penetration. Upon hydrolysis of the ester, MA2 yields MA within cells. Both MA and MA2 have been studied in cancer models. In mice engrafted with glioblastoma stem cells (GSCs), MA2 treatment significantly reduced tumour size, and prolonged survival, suggesting the therapeutic potential of increasing m6A level through FTO inhibition.^174^ Additionally, MA2 and the previously introduced Rhein were shown to restore nilotinib sensitivity in tyrosine kinase inhibitor (TKI) resistant leukaemia in vitro and in vivo.^175^ MA also reverses Gefitinib resistance in NSCLC cells, showing synergistic effects with Gefitinib in Gefitinib‐resistant NSCLC cells.^176^ Nevertheless, others have reported the tumour‐suppressive role of FTO,^177^ emphasising the need for further research before considering clinical studies involving FTO inhibitors in cancer.

Several competitive FTO inhibitors have been developed by optimising MA to enhance potency, target selectivity, and pharmacokinetics. Among all synthesised analogues, FB23 stand out with the highest potency, showing an approximately 140‐fold increase over MA.^178^ The derivative of FB23, named FB23‐2, demonstrates improved cellular permeability, leading to increased m6A levels and exhibits anti‐proliferative efficacy in various AML cell lines.^178^ Moreover, FB23‐2 inhibits AML progression in xeno‐transplanted mice, resulting in prolonged survival.^178^ The selectivity of MA is retained in these optimised inhibitors by preserving the benzyl carboxylic acid that interacts with the non‐conserved loop in FTO.^174^, ^178^

Subsequent optimisation of FB23 led to the discovery of Dac51 which forms additional hydrogen bonds with the FTO protein, improving potency.^144^ Considering FTO's role in the TME remodelling and its involvement in immune surveillance, co‐culturing Dac51‐pretreated B16OVA melanoma cells with T cells demonstrated enhanced cytokines release and elevated cytotoxic capacity.^144^ In vivo treatment with Dac51 increased the proportion of infiltrated CD8+ T cells in the TME and effectively inhibited tumour growth. Furthermore, combining Dac51 with an immune checkpoint blockade significantly prolonged the survival of mice compared to monotherapy.^144^

The design and synthesis of FB23 analogues, along with Structure–Activity Studies led to the discovery of compound 13a. It significantly inhibits FTO demethylation in vitro, suppresses AML cell proliferation, and improves the survival of MONOMAC6‐grafted mice without displaying apparent off‐target effects.^179^

Cao et al. enhanced the efficacy of MA in tumour cells using nanoparticle technology for targeted delivery.^180^ They developed GNCP12, a nanocluster with a 12‐mer peptide (GGGCDLRSAAVC), which specifically targets C‐type lectin‐like molecule‐1 that overexpressed on AML cells and CD34 + CD38+ leukaemic stem cells (LSCs). By incorporating GSH‐S‐DNP, a GSH derivative, as the imprinting template to create a hydrophobic pocket in the nanoparticle, GNCP12 binds the thiol group of GSH via ligand exchange in the hypoxic bone marrow. This triggers the selective release of loaded MA in the presence of GSH, enabling the targeted killing of AML cells and LSCs. Combining this nanoparticle therapy, termed GNPIPP12MA, with PD‐L1 blockade effectively inhibited leukaemia progression and metastasis in the preclinical mouse model.^180^

Huff et al. employed the binding pocket occupied by the selective MA to rationally design unique inhibitors while maintaining selectivity.^181^ They identified the pyrimidine scaffold as a promising replacement for the benzyl carboxylic group of MA, which provides the necessary selectivity. Subsequently, fragment growth was directed towards the unoccupied binding pocket, leading to the discovery of FTO‐4. FTO‐4 increases the m6A level of GSCs and impairs self‐renewal in GSC‐derived neurospheres.^181^ Further optimisation of FTO‐4 led to FTO‐43, which exhibits anti‐proliferative efficacy in multiple in vitro cancer models, including AML, glioblastoma, and gastric cancer.^182^

R‐2‐hydroxyglutarate (R‐2HG), a metabolite produced by mutant isocitrate dehydrogenases (IDHs), has been shown to inhibit FTO demethylation activity, leading to the downregulation of the oncogenic MYC.^183^ In xenografted mice experiments, both direct injection of R‐2HG and IDH1R132H‐mediated R‐2HG generation significantly inhibited AML progression, indicating therapeutic potential. Additionally, Qing et al. showed that R‐2HG inhibits glycolysis in AML by suppressing FTO's activity.^184^ This understanding sheds light on how R‐2HG may contribute to resistance to mutant IDH inhibitors. Consequently, combining a mutant IDH inhibitor with an FTO inhibitor like R‐2HG may hold therapeutic potential in treating resistant AML.

Saikosaponin‐D (SsD), a naturally occurring triterpenoid saponin found in the roots of *Bupleurum falcatum*, competitively inhibits the demethylation activity of FTO.^185^ Like MA, SsD has shown the ability to overcome FTO/m6A‐mediated leukaemia resistance to TKI.^185^ Notably, SsD has been used in traditional Chinese medicine due to its anti‐inflammatory and hepatoprotective properties, suggesting its potential safety.

#### 5.2.2 Other FTO inhibitors

FTO inhibitor MO‐I‐500 has been reported to inhibit FTO demethylation in vitro, but its precise mode of action remains unclear due to the lack of crystal structure.^186^ MO‐I‐500 significantly inhibits cell survival and colony formation of inflammatory breast cancer SUM149‐MA cells compared to untreated cells or those treated with an inactive analogue, MO‐I‐100.^187^ However, resistance developed with prolonged co‐culture in a glutamine‐free medium, suggesting potential adaptive mechanisms or cellular changes overcoming the inhibitor's effects. Moreover, this inhibitory effect was not observed when cells were cultured in a medium containing glutamine, indicating that metabolic stress may play a role in MO‐I‐500's activity.

CS1 and CS2 are potent FTO inhibitors with undisclosed modes of action.^188^ Both induce apoptosis and G0 phase cell cycle arrest in human cells. In a patient‐derived xeno‐transplanted AML model, CS2 treatment reduces leukaemia infiltration and doubles survival. On the other hand, CS1 only shows enhanced anti‐leukaemia activity when delivered in micelles.^188^ Furthermore, inhibition of FTO by CS1 or CS2 inhibits immune evasion in AML cells in vivo. More recently, Phan et al. demonstrated the in vivo anti‐tumour efficacy of CS1 in CRC.^189^

#### 5.2.3 FTO activators

Tricyclic antidepressants (TCAs) are among the first antidepressants developed.^190^ Imipramine (IMI) and Amitriptyline (AMI) activate FTO function and reduce m6A levels in N2a cells,^190^ potentially contributing to their antidepressant effects. However, further research is needed to investigate their anti‐tumour efficacy.

### 5.3 M6A eraser‐ALKBH5

#### 5.3.1 ALKBH5 inhibitors

ALK‐04 is a selective ALKBH5 inhibitor identified through in silico screening and the subsequent structure–activity relationship studies.^146^ Combining ALK‐04 with immunotherapy significantly reduces tumour growth in mice, suggesting its potential to overcome anti‐PD1 resistance and enhance immunotherapy effectiveness.^146^

MV1035, a sodium channel blocker, reduced the migration and invasiveness of U87 glioblastoma cells.^191^ Interestingly, the reference sodium channel blocker TTX did not produce similar results, indicating that the anti‐tumour effect is unrelated to sodium channel blocking. The study used SPILLO‐PBSS software to explore the underlying mechanism and identified potential off‐targets on a proteome‐wide scale. The result indicated that MV1035 competitively binds to the cofactor site of ALKBH5, leading to increased cellular m6A‐tagged mRNA and reduction of the oncoprotein CD73 expression.^191^

In virtual screening of 144,000 compounds from a library developed by the Institute for Molecular Medicine Finland identified 2‐[(1‐hydroxy‐2‐oxo‐2‐phenylethyl)sulfanyl]acetic acid and 4‐{[(furan‐2‐yl)methyl]amino}‐1,2‐diazinane‐3,6‐dione.^182^ In vitro experiments on leukaemic and glioblastoma cells showed their potential as selective anti‐proliferative agents for some cancer cell lines (i.e. HL‐60, CCRF‐CEM, and K562), but not for Jurkat or A172 cells. This highlights the complexity of m6A regulators' role in cancers, emphasising the need for further research to understand subtype‐specific functions.^182^

Compound 20 m is a potent and selective ALKBH5 inhibitor, stabilising ALKBH5 in human hepatoma cells. However, its anti‐proliferative effects in vitro or in vivo remain to be determined.^192^

Two compounds, Ena21 and Ena15, were discovered through the HTS of the Enamine Pharmacological Diversity Set.^193^ Docking studies revealed that Ena21 occupies the cofactor (2OG) binding site, suggesting it is a competitive inhibitor. However, Ena15 does not show such binding. Enzyme kinetic experiments support this conclusion. Inhibition of ALKBH5 with Ena21 and Ena15 successfully inhibits cell proliferation of glioblastoma multiforme‐derived cells and decreases cell population in the synthesis phase of the cell cycle.^193^

### 5.4 M6A reader‐IGF2BPs

#### 5.4.1 Allosteric inhibitor

HTS of ~16,190 compounds from three libraries (i.e. the ChemBridge MicroFormat, the Unversity of Illinois Marvel library, and the NCI Diversity Set) identified BTYNB.^194^ BTYNB specifically targets and inhibits cell proliferation of IGF2BP1‐positive cells but not IGF2BP1‐negative cells in melanoma and ovarian cancer cell lines. It also impairs cell proliferation and induces apoptosis in Neuroendocrine Neoplasm (NEN) cells.^195^ Testing on leukaemic cells showed decreased cell viability, increased cell death, and cell cycle arrest at S‐phase.^196^ In vivo, BTYNB shows promising anti‐tumour efficacy in xenograft models of intrahepatic cholangiocarcinoma and ovarian cancer.^197^, ^198^

CuB, identified from HTS of 889 compounds from the Medicinal Natural Products Library, allosterically binds IGF2BP1, altering expression of downstream RNA such as c‐MYC, Kras, and FSCN1.^199^ In vivo, CuB triggers apoptosis, recruits immune cells to the TME, and inhibits the expression of PD‐L1.^199^

#### 5.4.2 Competitive inhibitor

Inhibitors of IGF2BP2 have also been identified through HTS of ~1200 compounds (Table 8). Ten compounds were identified, including 4 benzamidobenzoic acid class and 6 ureidothiophene class compounds, which inhibits cell proliferation in CRC cells.^200^ Three compounds tested show significant anti‐tumour effects in a zebrafish xenograft model with minimal toxicity.^200^ However, poor membrane permeability limited their induction of cell death and a high dose is required. Virtual screening of 300,000 compounds via docking into the RNA‐binding site of IGF2BP2 followed by cellular assay identified the inhibitor JX5.^138^ JX5 shows cytotoxicity against Jurkat cells with IGF2BP2 overexpressed but only mild inhibition in normal Jurkat cells, suggesting therapeutic potential for IGF2BP2‐positive leukaemia. The anti‐leukaemic effect was also confirmed in vivo.

**TABLE 8:** Therapies targeting IGF2BPs.

m6A regulators	Mode of action	Therapy	Anti‐tumour effect (In vitro)	Anti‐tumour effect (In vivo)	Disease	References
IGF2BP1	Allosteric inhibitor	CuB (cucurbitacin B)	Induced apoptosis of HCC cells.	Exhibits anti‐HCC effect through inducing apoptosis and recruiting immune cells to the tumour microenvironment as well as blocking PD‐L1 expression.	HCC	[199]
		BTYNB	Inhibits cell proliferation of IMP1‐positive cancer cells		Melanoma, Ovarian Cancer	[194, 195, 196, 197, 198, 281]
				Exerts promising anti‐tumour efficacy in a patient‐derived xenograft intrahepatic cholangiocarcinoma model.	Intrahepatic cholangiocarcinoma	
			Impairs cell proliferation and induces apoptosis in NEN cells.		Neuroendocrin neoplasms	
					High‐risk neuroblastoma	
			Decreases cell viability of leukaemic cells while increases cell death and cell cycle arrest at S‐phase.		Leukaemia	
			Impairs viability of LUAD‐derived A549 cells.	Impairs the growth and spread of tumour cells with reduced tumour burden in ovarian cancer xenografted model.	Pancreatic ductal adenocarcinoma carcinogenesis, Potentiated lung adenocarcinoma (LUAD) cell, Ovarian cancer	
		7773	Inhibits cell migration of H1299, Es2, and HEK293 cell lines.		Lung cancer, ovarian cancer	[201]
IGF2BP2	Competitive inhibitor	4 benzamidobenzoic acid class and 6 ureidothiophene class	Inhibits tumour cell proliferation of HCT116, SW480, and Huh7 cells in 2D and 3D cultures.	Inhibits tumour growth with minimal toxicity (zebrafish model).	Colorectal cancer, lung cancer	[200]
		JX5	Shows cytotoxicity against Jurkat cells with IGF2BP2 overexpression.	Treated mice have prolonged survival with reduced human CD45+ cells in the bone marrow and spleen	T‐ALL	[138]
IGF2BP3	No direct target	Isoliquiritigenin (ISL)	Inhibits the expression of IGF2BP3.		NSCLC	[202]

#### 5.4.3 Other inhibitors

Another IGF2BP1 inhibitor identified via HTS is known as 7773.^201^ It binds a hydrophobic surface of IGF2BP1, inhibiting its binding to Kras RNA and other target RNAs. As a result, 7773 significantly reduces Kras expression and affects the downstream pathway, leading to a reduced pERK/ERK ratio. In vitro, 7773 inhibits cell migration in H1299, ES2, and HEK293 cell lines while cell proliferation remains unaffected.

To the best of our knowledge, no inhibitor of IGF2BP3 has been reported. However, Isoliquiritigenin, derived from the Chinese herb licorice, significantly reduces the expression of IGF2BP3. Downregulation of IGF2BP3 inhibits the downstream TWIST1 mRNA expression, consequently exhibiting an anti‐tumour effect in NSCLC.^202^

## 6 LIMITATION OF TARGETING m6A REGULATORS

All the candidates discussed exhibit the potential to modulate m6A regulators, with many demonstrating potency and specificity as inhibitors. However, the inherent complexity of m6A modification poses a potential limitation for the application of these inhibitors. Much evidence suggests that targeting m6A regulators can be tumour‐specific to some extent. An example is the overexpression of METTL3 in AML cells compared to healthy haematopoietic cells.^41^ Pharmacologically inhibiting METTL3 using STM2457 did not show adverse effects in the development of normal bone marrow cells.^153^ However, this remains to be seen in other cell types and in conditions whereby cells are exposed to stress or environments that can lead to adverse effects upon METTL3 depletion.

METTL3‐mediated m6A methylation increases the maturation of miR‐355, promoting stress granule (SG) formation and reducing the apoptosis level of injured neurons and cells in acute ischemic stroke.^203^ Therefore, targeting m6A regulators may not be applicable to all patients, and the risk of causing stroke needs to be studied carefully.

Moreover, knockdown of YTHDFs was also reported to reduce SG formation,^40^ potentially leading to adverse effects if inhibited. However, another study argues that m6A modification only explains 6% of the variance in SG localisation and that it plays a minimal, if any, role in mRNA partitioning in SG formation. Nonetheless, evaluating the importance of a biological pathway by how often it occurs can be quite biased, while the existence of its complementary process is underappreciated.

Another major argument within the field includes the opposing role of the m6A regulators in certain cancers, examples include the conflicting results published around breast cancer. The reason for the observed inconsistency is underappreciated, which demands further research. This inconsistency is evident not only in the contrasting roles of m6A regulators across different and same cancer subtypes but also in their potential opposing impacts on cancer cells and immune cells. In the past decade, immunotherapy has brought about a revolutionary shift in the field of cancer treatment, where immune cells play a crucial, if not determinative, role in patient prognosis. Therefore, it becomes imperative to acknowledge the intricacies involved in specifically targeting m6A regulators within the appropriate immune cell types.

In the future, there are some strategies we can possibly use: (1) single‐cell analysis of cell subsets: an in‐depth exploration at the single‐cell level is essential to unveil the specific functions of m6A regulators within different cell types and cancer types. This approach allows for a comprehensive understanding of the complex roles these regulators play in diverse cellular contexts; (2) development of efficient targeted delivery: advancements in biotechnology are necessary to enable the precise delivery of drugs; developing technologies that facilitate targeted delivery ensures that the impact of m6A modulation is concentrated in specific cell types, thus minimising unintended consequences, and enhancing therapeutic efficacy. (3) investigating the normal physiological role of m6A regulators: minimising side effects is crucial, especially when dealing with potentially adverse pathways associated with m6A mRNA modification.

## 7 CONCLUSIONS AND FUTURE DIRECTIONS

The complex roles of m6A regulators in cancer highlight the need for further research to unravel their subtype‐specific functions. The diverse landscape of m6A regulators and their involvement in tumorigenesis underscore the importance of understanding their context‐dependent roles in different cancer types. By investigating the subtype‐specific functions of m6A regulators, we can uncover valuable insights that may guide precision cancer therapeutics.

We have discussed the current understanding of m6A regulators and their implications in cancer pathology. In this review, we focused on the potential of targeting these regulators as a therapeutic strategy, showcasing various inhibitors that have shown promise in preclinical studies. However, to fully harness the therapeutic potential of compounds targeting m6A regulators, it is crucial to delve into their efficacy in specific cancer subtypes, and consider the effects on immune cells and normal cells which could potentially influence cancer progression and lead to adverse effects. This precision medicine approach will enable the development of targeted therapies that address the specific molecular aberrations within individual tumours.

## AUTHOR CONTRIBUTIONS

**Angel Guan:** Data curation (lead); writing – original draft (lead). **Justin J.‐L. Wong:** Conceptualization (lead); supervision (lead); writing – review and editing (lead).

## CONFLICT OF INTEREST STATEMENT

The authors declare no competing financial interest.

## Data Availability

Data sharing is not applicable to this article as no new data were created or analyzed in this study.
